# The effect of sewage sludge and BAF inoculant on plant condition and yield as well as biochemical and microbial activity of soil in willow (*Salix viminalis* L.) culture as an energy crop

**DOI:** 10.7717/peerj.6434

**Published:** 2019-03-11

**Authors:** Katarzyna Panasiewicz, Alicja Niewiadomska, Hanna Sulewska, Agnieszka Wolna-Maruwka, Klaudia Borowiak, Anna Budka, Karolina Ratajczak

**Affiliations:** 1 Department of Agronomy, Poznań University of Life Sciences, Poznań, Poland; 2 Department of General and Environmental Microbiology, Poznań University of Life Sciences, Poznań, Poland; 3 Department of Ecology and Environmental and Protection, Poznań University of Life Sciences, Poznań, Poland; 4 Department of Mathematical and Statistical Methods, Poznań University of Life Sciences, Poznań, Poland

**Keywords:** Phosphomonoesterase, Bacteria, Dehydrogenases, Photosynthesis activity, Willow

## Abstract

Excessive amounts of sewage sludge produced in sewage treatment plants along with the ban on its storage and dumping require rapid solutions to the problem of sewage sludge management. An example of a rational and environmentally viable method may be provided by its application in agriculture and environmental management. The optimal solution is to use sludge as a fertiliser for industrial plants, including energy crops, that is, those not used in food production. For environmental reasons it is essential to control soil quality and condition following sludge application. Analyses of the residual effect of sewage sludge and bacteria, actinobacteria, fungi microbial inoculant (BAF) on selected physiological parameters of plants and microbial activity of soil were conducted in the years 2013–2015 on experimental fields of the Poznan University of Life Sciences. The results indicate that the application of sewage sludge increased yields and improved selected photosynthesis activity and biometric traits of willow. Among the tested combinations the best results were obtained following the application of sewage sludge combined with the BAF medium microbial inoculant. Similar dependencies were observed when evaluating soil microbial activity.

## Introduction

It is predicted that the total amount of sewage sludge produced in the EU may reach 13 million tons in 2020. For this reason we need to search for rational methods of its management (Wołejko et al., 2014). Due to it being a potential environmental hazard and its physical and chemical properties, sewage sludge has to be subjected to appropriate processing (Pająk, 2014). Recently the last stage of sewage sludge processing consisted of mechanical dewatering followed by its storage. The ban on storage of municipal sewage sludge, imposed by legal regulations since January 2016, has had a significant impact on the Polish sewage sludge management system. Thus, it seems absolutely necessary to find appropriate management methods, which constitutes a considerable ecological and economic problem (Bień et al., 2011). Numerous reports indicate that, due to its properties, sewage sludge may be used to enrich and reclaim biologically degraded soils (Wierzbicki, 2003). An advantage of sewage sludge is connected with its contents of nutrients, primarily nitrogen and phosphorus compounds, as well as carbon compounds. Sewage sludge has to be thoroughly tested before it may be used for any purpose affecting the environment. It is safest to apply it as a fertiliser for industrial or energy crops, as they are not used to produce food. An example in this respect may be willow, which is not only a good source of biomass, but also a good phytoremediator, that is, capable of removing hazardous substances from the contaminated environment (Abhilash et al., 2012; Ali, Khan & Sajad, 2013). Moreover, the use of sewage sludge in fertilisation of soils to cultivate energy crops, including willow, is not only one of the more efficient disposal methods for this waste (Stańczyk-Mazanek, Bień & Kępa, 2014; Quaye & Volk, 2013), but is also economically profitable. Moreover, in view of the large proportion of light soils in Poland (60.8%), in comparison to the average EU proportion of 31.8%, sewage sludge management in plant production is recommendable in our country.

Also energy production from renewable sources is increasing rapidly due to concerns about energy security and climate change. Therefore, a transition towards the achievement of sustainable energy production systems (energy produced with minimal negative impacts on human health and healthy functioning of vital ecological systems, including the global environment) is of the utmost importance. In this sense, the European Union set legally binding targets through Directive 2009/28/EC, in order to reach a 20% share of renewable energy by 2020. Accordingly, energy from biomass will increase considerably by 2020 and it is expected to be partly fulfilled by dedicated energy crops cultivated in abandoned or marginal lands (Bentsen & Felby, 2012). The pedosphere should be considered in energy crop production systems. In this regard, one of the most important issues to address in order to protect soils is the soil organic matter content (Commission of the European Communities, 2006).

Soil microflora is a reliable indicator of the quality and condition of the soil environment, since microorganisms determine the equilibrium within the soil medium and ensure its adequate fertility. Application of microbiological indexes in analyses of the soil environment makes it possible to evaluate the ecological condition of soils. A significant problem in this respect is connected with assessment of the long-term effect of sewage sludge on functional diversity of microbial communities in the soil environment (Smalla & Van Elsas, 2010).

The aim of this study was to assess the effect of application of sewage sludge, the bacteria, actinobacteria, fungi microbial inoculant (BAF) as well as sewage sludge combined with the BAF on growth, photosynthesis activity and yield of willow as an energy crop, as well as the biochemical and microbial activity of soil in such energy crop willow culture. An interesting aspect was connected both with the direct effect of tested combinations (in the year of their application) and the residual effect in the two successive years of cultivation of this crop.

## Materials and Methods

### Experimental design and sample collection

Analyses of the residual effect of sewage sludge from the wastewater treatment plant in Szamotuły and the BAF inoculant on selected plant physiological parameters and soil microbial activity were performed in the years 2013–2015 on fields of the Experimental and Teaching Station in Gorzyń, branch in Złotniki (N: 52° 29′ 0″, E: 16° 49′ 53″), belonging to the Poznań University of Life Sciences (PULS). In the wastewater treatment plant in Szamotuły according to the Directive (91/271/EWG) of the purification process is used biological treatment of secondary sludge. In addition, the chemical process is used—the simultaneous removal of phosphorus with the help of PIX-113 coagulant. The wastewater treatment plant is not additionally supplied with industrial sewage. People Equivalent is on the level 28,880.

Two types of set of parameters were tested simultaneously during the experiment. One concerning the transformation of carbon in the soil after the application of sludge (presented here) and the second concerning the transformation of nitrogen. The latter one regarding nitrogen transformation was published by Wolna-Maruwka et al., 2018. Field trials were conducted as unifactorial experiments in four replications. Four variants were applied in the experiment: (1)—control (soil with no fertiliser application); (2)—soil fertilised with sewage sludge; (3)—soil fertilised with sewage sludge and inoculated with BAF; (4)—soil inoculated with BAF.

Willow (*Salix viminalis* L.) cv. Start was grown after winter rape on lessive soil formed from light loamy sands, classified in quality classes IVa and IVb, of the very good and good rye complex According to the World Reference Base this soil is classified among Albic Luvisols. In the autumn before the experiment was established a single mineral phosphorus–potassium fertilisation was applied by broadcasting phosphorus fertiliser (triple superphosphate, 46% P_2_O_5_) at 80 kg P_2_O_5_·ha^−1^ and potassium fertiliser (60% potassium salt) at 100 kg K_2_O·ha^−1^. Both macronutrients were used to provide plant nutrition during the first year of experiment and in subsequent years. In the spring prior to willow planting nitrogen fertilisation was applied using 34% ammonium nitrate at 90 kg N·ha^−1^. Stem cuttings 20 cm in length were planted on 25 April 2013 at a 70-cm row spacing and plant spacing of 35 cm within the row. Weeds were controlled mechanically when willow plants reached a height of 10–15 cm. No disease or pests were reported on plants during the vegetation period. The harvested plots were 15 m^2^ in size. The other cultivation measures were performed following the principles of good cultivation practice for that species.

In each year of culture after completion of the vegetation period plant number per unit area was determined; it did not change significantly in the period of the study. Moreover, shoot diameter at a height of 30 cm from the ground, plant height (PH) and the number of shoots (NSs) from the rootstock were determined on five successive plants. The yield of biomass was assessed each time after cutting all shoots from the plot using a rotating disc mower. Moisture content of the harvested material was determined by the dry-weight method.

### Characterization of sewage sludge

In the above-mentioned variants sewage sludge was applied to the soil once in the spring of 2013 (March), and then analyses of the application effects were performed three times in each of the years of analysis (May, July, September). The sewage sludge application rate per one m^2^ was 0.00451 Mg d.m., and it did not exceed the dose of municipal sewage sludge for reclaimed areas and those cropped for non-food or non-feed purposes admissible under the Regulation of the Minister of Environmental of 13 July 2010, specifying it at a total 45 Mg d.m.·ha·3 years^−1^. Characteristics of the basic chemical composition of soil prior to sludge application and after its application are given in Table 1. Sludge used in the experiment met the requirements of respective standards in relation to contents of heavy metals and biological contamination.

**Table 1:** Chemical characteristics of soil.

Specification	Control soil	Soil with sewage sludge	Threshold values
pH KCl	6.5	6.7	–
Macroelements			
Tot-N % d.m.	0.02	0.02	–
P_2_O_5_ mg·100g^−1^ soil	22.5	25.2	>22
K_2_O mg·100g^−1^ soil	13.6	18.6	12,6–20
CaO % d.m.	0.09	0.10	–
Mg mg·100g^−1^ soil	4.2	4.2	3,1–5,0
Microelements (mg·kg d.m.^−1^)			
Pb	7.5	8.8	40
Cd	0.160	0.173	1
Ni	4.37	4.97	20
Hg	0.022	0.022	0.8
Zn	23.3	25.0	80
Cu	7.3	7.3	25
Cr	10.00	10.00	50

### Characterization of microbial consortium

In turn, the applied BAF, applied directly to soil in experimental variants three and four, was developed at the Department of General and Environmental Microbiology, PULS.

The biopreparation consisted of 15 strains of bacteria (*Bacillus subtilis, B. amyloliquefaciens*), five of actinomycetes (*Streptomyces* spp.) isolated from mature compost prepared from plant residues and sewage sludge as well as four strains of *Trichoderma harzianum* fungus derived from the collection of the Institute of Plant Genetics in Poznań.

The above-mentioned strains were examined, among others, from the point of view of their proteolytic and cellulolytic activities. The inoculant was tested in terms of its biochemical activity. The bacterial count in one ml of inoculant was 1.76·10^6^ cfu, the count of Actinobacteria was 2.3·10^3^ cfu, while that of fungi was 1.89 10^2^ cfu.

### Photosynthesis activity

The *C*_i_ 340aa handheld photosynthesis system (CID BIOSCIENCE Inc., Camas, WA, USA) was used to evaluate net photosynthetic rate (*P*_N_), stomatal conductance (*g*_s_), transpiration rate (*E*) and intercellular CO_2_ (*C*_i_) concentration. For these purposes constant conditions were maintained for leaf chamber measurements: CO_2_ inflow concentration (390 μmol CO_2_) mol^−1^, photosynthetic photon flux density 1,000 μmol (photon) m^−2^·s^−1^, chamber temperature 25 °C, relative humidity 40 ± 3%. These measurements were performed three times during the experiment in the 3rd, 15th and 27th month, respectively, 1st, 2nd, 3rd year. For this purpose five plants of each combination were selected.

### Microbiological and biochemical analyses

Soil samples for biochemical and microbiological analyses in each vegetation season were collected at three dates (May, July and September). In soil samples collected from a depth of 0–20 cm under plants microbial counts were determined.

Microbiological analysis was performed on the basis of serial dilution method and involved determining (using selective substrate in five replications) the numbers of colony forming units (CFU g^−1^ d.m. of soil) of the total number bacteria, oligotrophic and copiotrophic bacteria, Actinobacteria, and fungi. Estimation of CFU number of the above-mentioned microorganisms is a measure of the intensity of their current metabolic activity.

The total bacterial number was determined on Merck standard agar (three g yeast extract; 5.0 g peptone from casein (free from fermentable carbohydrates); five g sodium chloride; 12 g agar, one l H_2_O), after 5–6 days of incubation at 28 °C. Oligotrophic bacteria were counted on diluted nutritive broth (0.1 g peptone, 0.1 g beef extract, 0.05 g sodium chloride, 20 g agar, one l H_2_O) at 28 °C after 21 incubation days (Ohta & Hattori, 1980). Copiotrophic bacteria were determined on nutritive broth (10 g peptone, 10 g beef extract, five g sodium chloride, 20 g agar, one l H_2_O) at 28 °C after 7 days of incubation (Ohta & Hattori, 1980). Fungi were determined on Martin substrate (one g KH_2_PO_4_, 0.5 g MgSO_4_, five g peptone, 10 g glucose, 3.3 ml Bengal, 0.1 g chlortetracycline, 25 g agar, one l H_2_O) for 5 days at 24 °C (Martin, 1950), and numbers of Actinobacteria were assessed on a selective Pochon substrate (0.05 g asparagine, 0.1 g nystatin, two g starch, five g K_2_HPO_4_, 2.5 g MgSO_4_·7H_2_O, 2.5 g NaCl, 0.05 g MnSO_4_·5H_2_O, 0.05 g Fe_2_(SO_4_)_2_·5H_2_O, 25 g agar, one l H_2_O) following plate incubation for 7 days at 26 °C (Grabińska-Łoniewska, 1999).

The analyses of soil enzymatic activity in different variants were based on the spectophotometric method applied to measure dehydrogenases activity (DHA), where 1% TTC (triphenyltetrazolium chloride) was used as a substrate. The measurement took place after 24-h incubation at a temperature of 30 °C and a wavelength of 485 nm and it was expressed in μmol TPF (triphenyl formazan)·24 h^−1^ g^−1^dm of soil (Thalmann, 1968).

Additionally, biochemical analyses of soil involved the determination of activities of acid phosphomonoesterase (PAC) (EC 3.1.3.2) and alkaline phosphomonoesterase (PAL) (EC 3.1.3.2.) according to Tabatabai & Bremner (1969). The activities were determined using disodium p-nitrophenyl phosphate tetrahydrate as a substrate, after 1 h incubation at 37 °C at a wavelength of 400 nm. Results were converted into μmol (p-nitrophenol) PNP h^−1^ g^−1^ d.m. of soil. A pH-appropriate buffer was used to determine each of the phosphatases.

### Weather conditions

It is generally accepted that climatic conditions found throughout Poland are suitable for willow cultivation. This species needs approx. 500 mm annual precipitation to grow. Weather conditions in the years of the study during the vegetation period of willow are presented using the hydrothermal index according to Sielianinov (Table 2). The hydrothermal index Sielianinov expressed as a quotient of the amount of the monthly sum atmospheric precipitation and sum of average daily air temperatures in a given month for a period of time. In all the years mean air temperature ranged from 8.6 to 10 °C and it was comparable to the multiannual mean. A crucial element of optimal growth and biomass yielding conditions is also total precipitation, particularly its distribution. Variability of weather conditions in the years of the study was reflected in the values of the Sielianinov index. More advantageous moisture conditions for willow plants were found in the years 2013 and 2014 than in the drier 2015 (*K* = 0.9), which was reflected in the volume of produced biomass.

**Table 2:** Sielianinov hydrothermal coefficient (K) according to weather conditions from April to September at Experimental Station Złotniki in 2013–2015.

Year	Months							Average
	IV	V	VI	VII	VIII	IX	X	
2013	0.72	1.81	2.04	0.76	0.76	2.00	0.52	1.23
2014	1.81	2.25	0.89	0.70	1.68	1.01	0.40	1.25
2015	0.90	1.10	0.57	0.91	1.24	0.84	0.93	0.92
1951–2015	1.14	1.72	1.16	0.79	1.39	1.28	1.16	1.14

Interpretation of the Sielianinov hydrothermal coefficient:

*K* > 1.5—excessive moisture for all plants
*K* = 1.0–1.5—sufficient moisture
*K* = 0.5–1.0—insufficient moisture
*K* < 0.5—moisture level below the requirement for most plants—drought.

### Statistical analysis

Statistical evaluation were performed using the Microsoft Excel and Statistica 12.0 (StatSoft Inc., Krakow, Poland) software packages. In order to compare mean values of the biological parameters at individual dates of analysis and the effect of sewage sludge the two-way analysis of variance was used (α = 0.05). It was followed by detailed Tukey’s test (post-hoc Tukey HSD).

To present results of microbiological analyses in order to select the multiple regression model, stepwise regression with the backward sequence procedure from the 5th degree was applied. Independent variables were successive powers of the variable X as time from the application of sewage sludge and supplemented with the BAF, while the counts of selected microbial groups and soil enzymatic activity were the dependent variables. Moreover, the coefficient of determination *R*^2^ was used to define the goodness of fit of the model.

The data were subjected to a principal component analysis (PCA). The results of PCA are presented in graphic form. Pearson correlation analysis was carried out for all analysed variables. These analyses were performed for dry matter yield (DMY), shoot thickness (ST), PH, and NS.

Two heatmaps and cluster analyses were here performed—the first one analysed similarities between the microbial soil activity, gas exchange parameters and terms of analysis and experimental variants. The second one investigated the correlations between variables. The first one presents mean values of measured parameters at a certain term of analysis. Afterwards a cluster analysis was performed to group similar terms and variants in regard to all analysed parameters. Euclidean distance measures and Ward hierarchical clustering were used to determine the dendrogram. The Euclidean distance measure can designate a similar structure in interactions between analysed parameters.

In the second heatmap, Pearson’s correlation matrix was designated for the soil microbial activity and gas exchange parameters. Afterwards, a cluster analysis was performed, in order to arrange analysed parameters in groups, with the highest degree of association within each group and the lowest degree of association between different groups.

## Results

### Soil microorganisms

Statistical analyses were conducted for the results of 3-year studies on the residual effect of sewage sludge on counts of selected groups of soil microorganisms under energy willow culture. The two-way analysis of variance showed a highly significant effect (α = 0.001) of the investigated factors on the dynamics of changes in the counts of selected groups of soil microorganisms, such as the total bacterial count, counts of Actinobacteria, oligotrophic and copiotrophic bacteria (Table 3). Multiple regression analysis showed that best models for each tested microbiological parameter (counts of selected microbial groups and microbial activity) were never higher than 2nd degree models. No model of a higher degree showed a better goodness of fit, while determination of coefficient *R*^2^ for the 2nd degree model was the highest, ranging from approx. 0.2 to 0.8.

**Table 3:** *F*-test statistics and significance levels of two-way analysis of variance for the number of selected groups of microorganisms associated with sewage sludge and years research as fixed factors.

Parameter	Year	Treatment	Interaction
Total number bacteria	112.0350***	3.81***	16.3***
Fungi	32.54***	17.27***	3.83***
Actinobacteria	615.65***	144.79**	30.55***
Copiotrophic	76.75***	7.315***	2.29***
Oligotrophic	445.29***	25.70***	2.02***

**Notes:**

ns, not statistically significant.

** *P* = 0.01.

*** *P* = 0.001.

**Figure 1:**
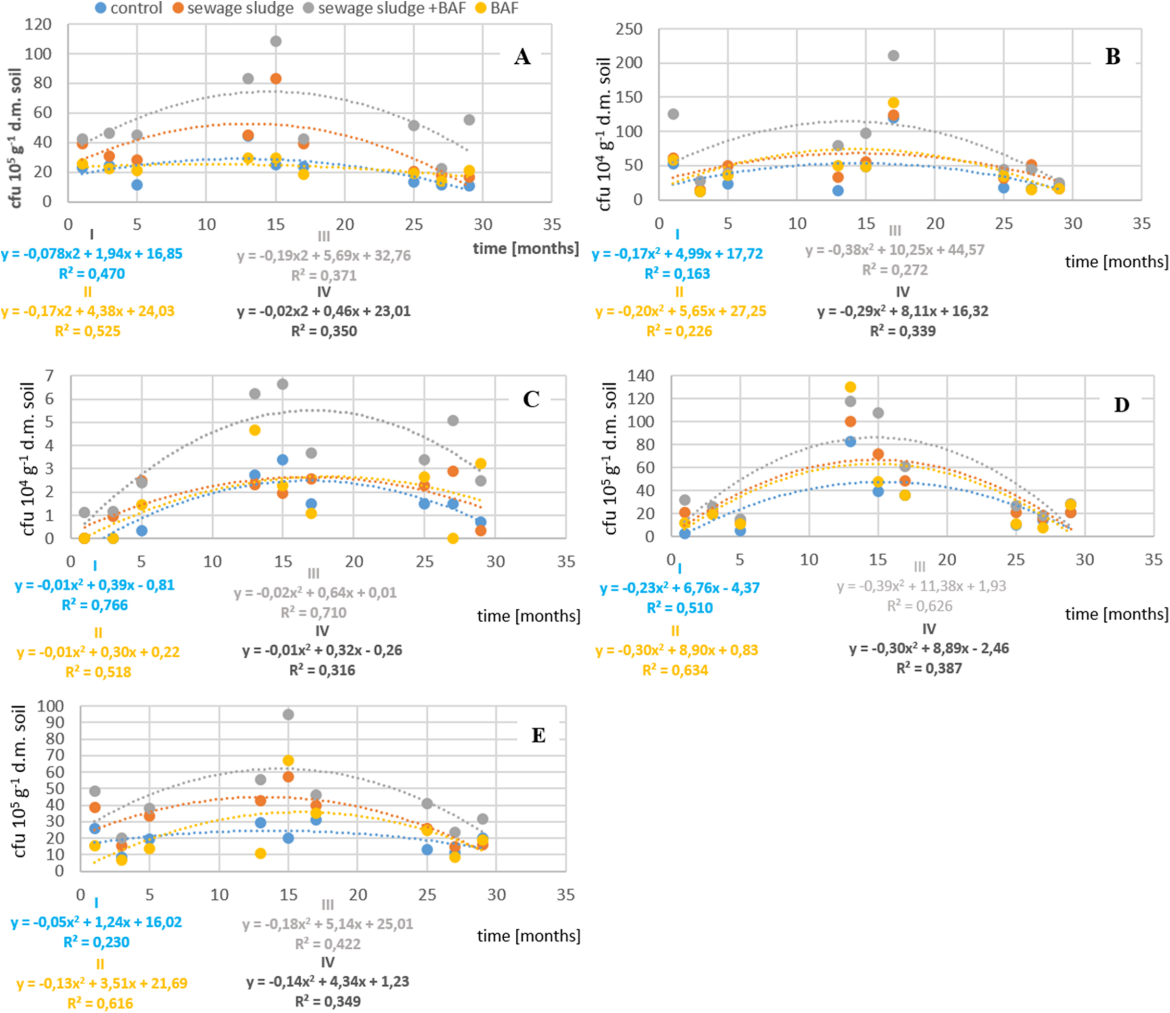
Impact of sludge sewage on the number of microorganisms. (A) Impact of sewage sludge and BAF on total number of bacteria. (B) Impact of sewage sludge and BAF on number of Actinobacteria. (C) Impact of sewage sludge and BAF on number of fungi. (D) Impact of sewage sludge and BAF on number of oligotrophic bacteria. (E) Impact of sewage sludge and BAF on number of copiotrophic bacteria.

The analysis of mean results from a given year for individual experimental variants showed that the application of sewage sludge and sewage sludge combined with the BAF modified the total bacterial counts in the soil both in the year of application and in the next year (Fig. 1A). The highest total number of microorganisms was recorded in the second year of sludge influence in the variant in which sludge was applied in combination with the BAF. In the third year of analysis, counts of the microbial group decreased in all experimental variants; nevertheless, the highest count was recorded in the same treatment as in the second year. Analogously as in the case of the total number of bacteria, analyses of mean counts of Actinobacteria from a given year in individual treatments indicate a significant stimulatory effect of applied municipal sludge combined with the BAF (Fig. 1B). In the other experimental variants when interpreting the results for the first, second and third year, the number of that microbial group was comparable to that in the control.

Data presented in our results show that municipal sewage sludge and the application of the BAF, as well as the time from their application, had a significant effect on the fluctuations in counts of fungi in the soil. Analyses of mean results from a given year for individual experimental treatments (Fig. 1C) showed significant stimulation of fungal growth by sewage sludge in the second year of field experiments in the treatment in which sludge and the BAF were applied simultaneously.

Analysis of mean counts of oligotrophic bacteria in a given year for individual experimental treatments showed that the growth of investigated microorganisms was stimulated by sewage sludge introduced to the soil only in the second year of this waste impact (Fig. 1D). In turn, growth of another microbial group, that is, copiotrophs, was stimulated by sewage sludge introduced to the soil in all the years of impact of this waste. Similarly as in the case of other microbial groups, this effect was most markedly reflected also in the second year of sludge impact in the variant in which sludge was applied together with the BAF, while it was weakest in the third year, as presented in Fig. 1E.

### Biochemical activity

The two-way analysis of variance showed a highly significant effect (α = 0.001) of investigated factors on the activity of selected soil enzymes: dehydrogenases and acid and alkaline phosphatases (Table 4).

**Table 4:** *F*-test statistics and significance levels of two-way analysis of variance for the activity of selected enzymes associated with sewage sludge and years research as fixed factors.

Parameter	Year	Treatment	Interaction
Dehydrogenases	33.71***	91.41***	9.45***
Acid phosphatase	112.08***	16.05***	9.18***
Alkaline phosphatase	76.78***	27.29***	12.85***

**Notes:**

ns, not statistically significant.

*** *P* = 0.001.

**Figure 2:**
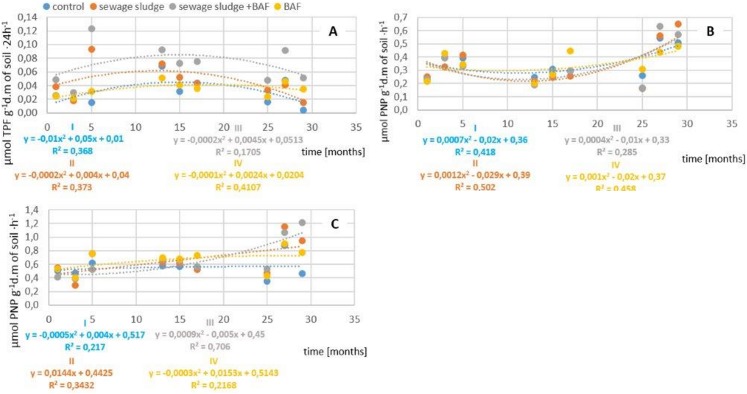
Impact of sludge sewage on biochemical activity. (A) Impact of sewage sludge and BAF on dehydrogenases activity. (B) Impact of sewage sludge and BAF on acid phosphatase activity. (C) Impact of sewage sludge and BAF on alkaline phosphatase activity.

Dehydrogenase activity is considered as an indirect indicator of microbial counts and activity in the soil. The presented mean values of dehydrogenase activity from a given year over the period of three years following the application of sewage sludge and the BAF (Fig. 2A) indicated that the applied factors had a significant effect modifying levels of these enzymes in lessive soil. Similarly as in the case of counts of selected microbial groups, the highest level of dehydrogenase activity was recorded in the second year after the application of sewage sludge together with the BAF and sludge alone. Moreover, a strong stimulatory impact of sludge and the BAF was found not only in the second year of analysis, but also in the year of application and in the third year after application, with this activity in individual years exceeding that in the control by 63, 42 and 54%, respectively.

Phosphatases PAC (acid phosphatase) and PAL (alkaline phosphatase) were other enzymes used as indicators of soil quality in this experiment. The activity of acid phosphatase differed from that of alkaline phosphatase. In the variants in which sewage sludge and the BAF were applied, lower values were recorded for the activity of PAC in comparison to those in the control (Fig. 2B).

Results obtained in the field trials indicate the highest values of PAL in all the variants in which sewage sludge and the BAF were applied (Fig. 2C). Additionally, a stimulatory effect of sewage sludge on the activity of this enzyme was also recorded in the third year after its application.

### Estimation of photosynthesis activity

**Figure 3:**
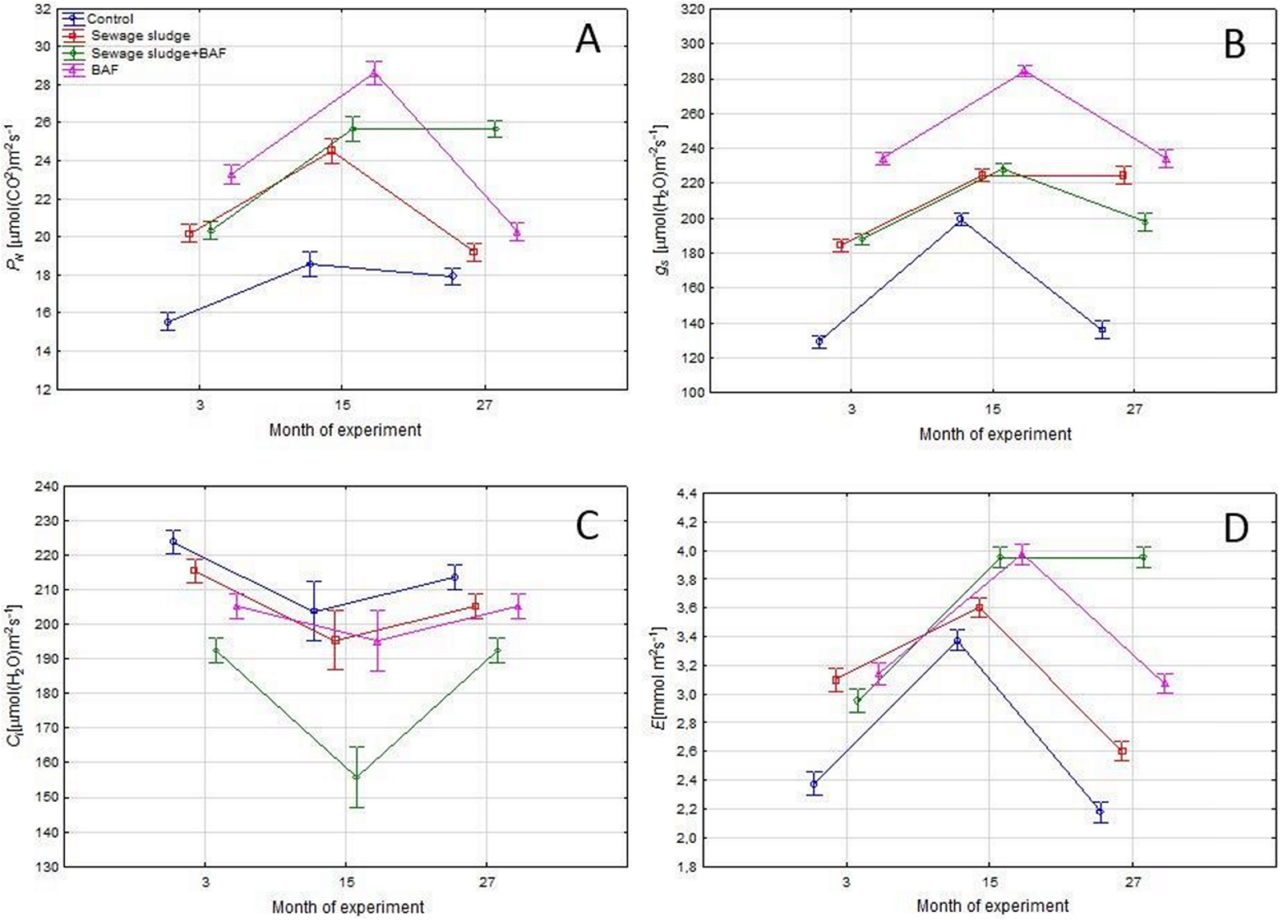
Gas exchange parameters (means ± SE) net photosynthesis rate (A), stomatal conductance (B), intercellular CO_2_ concentration (C) and transpiration rate (D) in 3rd, 15th and 27th month of experiment.

A highly significant (α ≤ 0.05) effect of date and variant of application on all analysed gas exchange parameters was found. An increase of net photosynthesis rate was noted for each variant in the 15th month of the experiment. However, the highest values were each time observed in the variant for used alone BAF, while the lowest level was recorded for control plants. At the 27th month of the experiment a decrease was noted to a similar level as at the beginning of the experiment. However, where sewage sludge and BAF and alone BAF were used, the values were highest at this measurement. A high level of net photosynthesis rate (*P*_N_) was related to stomatal conductance (*g*_s_) and transpiration rate, while intercellular CO_2_ concentration was the lowest in plants in the 15th month for plants with BAF application. There was a similar level of CO_2_ intercellular concentration (*C*_i_) in plants cultivated in the variants with sewage sludge and BAF and alone sewage sludge (Fig. 3).

**Figure 4:**
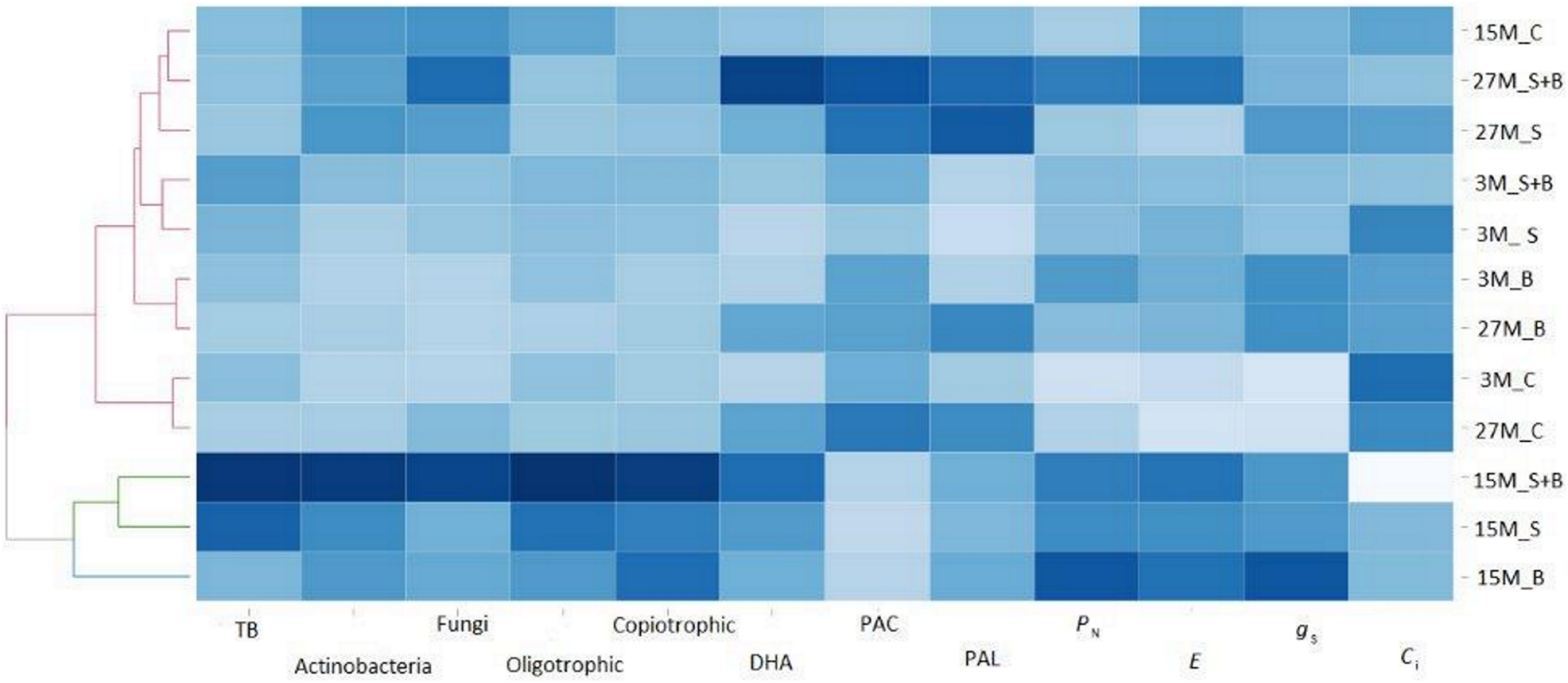
Comparable reaction between microbiological activity and gas exchange parameters subsequent in the months following the application of sludge sewage and sewage sludge with BAF. Abbreviation: TB, Total number bacteria; DHA, dehydrogenases activity; PAC, acid phosphomonoesterase; PAL, alkine phosphomonoesterase; *P*_N_, net photosynthesis rate; *E,* transpiration rate; *g*_s_, stomatal conductance; *C*_i_, CO_2_ intercellular concentration; 15M_C, fifteen month—control; 27M_S+B, twenty seven month after application sewage sludge and BAF inoculant; 27M_S, twenty seven month after application sewage sludge; 3M_S+B, three month after application sewage sludge and BAF inoculant; 3M_S, three month after application sewage sludge; 3M_B, three month after application BAF inoculant; 27M_B, twenty seven month after application BAF inoculant; 3M_C, three month—control; 27M_C, twenty seven month—control; 15M_S+B, fifteen month after application sewage sludge and BAF inoculant; 15M_S, fifteen month after application sewage sludge; 15M_B, fifteen month after application BAF inoculant.

The heatmap of interactions between mean values of microbial soil activity and gas exchange parameters revealed similarities between variants of sewage sludge, BAF and sewage sludge and BAF in the 15th month of experiment. The control was combined into one group for the 3rd and 27th month after application. The rest of variants and terms were grouped together. Based on this heatmap we can also find the lowest and highest values of the parameters. The highest mean values of bacteria, Actinobacteria, fungi and copiotrophic were recorded in the variant of sewage sludge and BAF in the 15th month after application, and in DHA and PAC in the 27th month after application of sewage sludge and BAF. PAL revealed the highest level in the 27th month in variant where alone sewage sludge, while *P*_N_ and *g*_s_ was noted for variant with BAF in the 15th month after application. We can also find that the highest parameters of soil microbial activities were observed together with high levels of *P*_N_, *g*_s_ and *E*, while *C*_i_ was recorded at the lowest level (Fig. 4).

**Figure 5:**
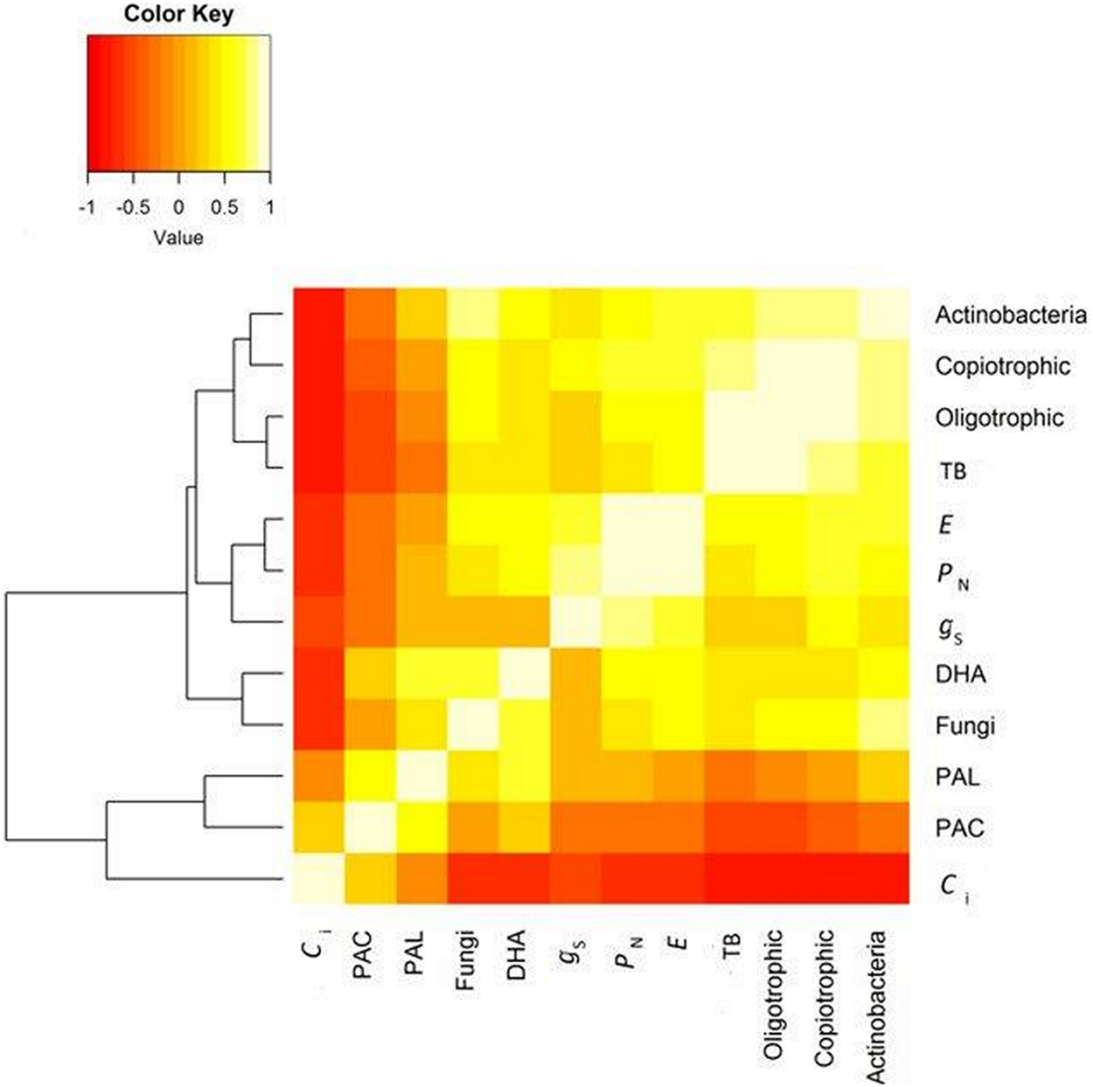
Correlation between gas exchange and all microbial parameters. Abbreviation: TB, Total number bacteria; E, transpiration rate; PN, net photosynthesis rate; *g*_s_, stomatal conductance; DHA, dehydrogenases activity; PAL, alkine phosphomonoesterase; PAC, acid phosphomonoesterase; *C*_i_, CO_2_ intercellular concentration.

A high negative correlation between *C*_i_ and all microbial parameters (excluding PAC and PAL) and *P*_N_, *g*_s_ and *E* was found. A negative relation of PAC and PAL with the groups of microorganisms and gas exchange parameters (excluding *C*_i_) was recorded. A positive relation of *P*_N_, *g*_s_ and *C*_i_ to the groups of microorganisms and DHA was found (Fig. 5).

### Biomass yield and biometric measurements

The analysis of variance for recorded results showed a significant effect of the years of analysis, the experimental factor as well as the interaction of the year of analysis and the variant of sludge application on the DMY, ST, and additionally a significant effect of the years of analysis on the NS and height of willow plants (PH) (Table 5).

**Table 5:** *F*-test from analysis of variance of treatment application on willow in years of study (2013–2015).

Source of variation	Dry matter yield	Thickness of shoot	Number of shoot	Height of shoot
Y	504.33**	82.869**	150.243**	39.057**
T	46.49**	9.706**	2.174 ns	1.968 ns
Y×T	19.85**	2.593*	1.816 ns	0.584 ns

**Notes:**

ns, not significant at 0.05 probability level.

Y, years.

T, treatment.

* significant at 0.05 probability level.

** significant at 0.01 probability level.

The yield of willow dry mass significantly varied in the years of the study (Table 6). A lower biomass was produced in the first year of culture, which is a characteristic trait of this species. Among the three analysed years the greatest DMY was recorded in the second year of the experiments. In the first year of culture the highest DMY was recorded in the treatment with sludge application (14.6 t·ha^−1^), while it was lowest in the control (11.3 t·ha^−1^). In 2014 the lowest yield was also found in the control (19.3 t·ha^−1^) and following the application of the BAF (19.2 t·ha^−1^), while it was highest after the combined application of sludge and the BAF (25.8 t·ha^−1^). In the third analysed year of the study, similarly as it was in the earlier years, the lowest yield of biomass was obtained in the control plot, with the highest yield harvested in the treatment with the combined application of sludge and the BAF.

**Table 6:** Dry mass and growth parameters of willow.

Year	Factor				Average
	Control	Sewage sludge	Sewage sludge + BAF	BAF	
Dry mass yield of willow (t·ha^−1^)					
2013	11.30 f g	14.65 f	12.05 g	12.63 f g	12.66 C
2014	19.26 d e	23.98 a b	25.82 a	19.16 d e	22.06 A
2015	17.90 e	19.63 d e	21.95 b c	21.00 c d	20.12 B
Average	16.39 C	19.77 A	20.39 A	17.72 B	–
Thickness of willow shoot (mm)					
2013	15.30 b c d	16.03 b c	15.85 b c	16.98 ab	16.04 B
2014	16.46 b	19.38 a	15.64 b c	16.74 b	17.06 A
2015	11.20 e	13.78 c d e	11.70 e	12.63 d e	12.33 C
Average	14.48 B	16.62 A	14.49 B	15.55 AB	–
Number of willow shoots (numer per plant)					
2013	1.68 a	1.80 a	2.10 a	1.95 a	1.88 B
2014	5.20 a	6.16 a	5.96 a	7.08 a	6.10 A
2015	5.52 a	5.70 a	6.35 a	5.45 a	5.76 A
Average	4.22 A	4.68 A	4.89 A	5.00 A	–
Height of willow shoot (m)					
2013	1.53 c d	1.88 b c	2.60 a	1.65 c d	1.91 B
2014	1.20 d	1.92 b c	2.50 a	1.48 c d	1.78 B
2015	1.83 b c	2.63 a	2.50 a	2.33 a b	2.32 A
Average	1.49 D	2.12 B	2.53 A	1.79 C	–

**Note:**

Means followed by different letters, capital letters and lower case letters are significantly different (Tukey’s test, *p* < 0.05).

In comparison to the control in the treatment with the combined application of sewage sludge and the BAF an increase in yield of willow dry mass was recorded, amounting to 3.7 t·ha^−1^. In contrast, no significant difference was found between treatments in which either sludge (19.4 t·ha^−1^) or sludge together with the BAF was used (19.9 t·ha^−1^). The smallest increment in the DMY in relation to the control, amounting to 1.4 t·ha^−1^, was recorded following the application of the BAF.

A significant effect on the years of the study and sludge application was also observed in the case of ST (Table 6). The thickest rods, with the diameter of 17.0 mm, were obtained in the second year of the study, while they were thinnest in the third, the driest year of culture (12.3 mm). In relation to the control the greatest increment in rod thickness (2.1 mm) was recorded in the sewage sludge treatment. In the first year of the experiments the thickest rods were obtained in the treatments in which the BAF was introduced to the soil, while in the following 2 years it was in treatments with sewage sludge application. The number of willow shoots recorded in this study was significantly modified by the year of culture (Table 6). The greatest NSs from a rootstock was obtained in the second year of the experiments, while no difference was observed in this respect between 2014 and 2015. The greatest, although not confirmed statistically, NSs was recorded in treatments with the application of the BAF. Analogously, year of culture had a significant effect on shoot height analysed in this study. The tallest plants were reported in the third year of analysis (Table 6). The tested combinations showed no effect on the value of this trait, with an upwards trend for height observed following each application.

**Figure 6:**
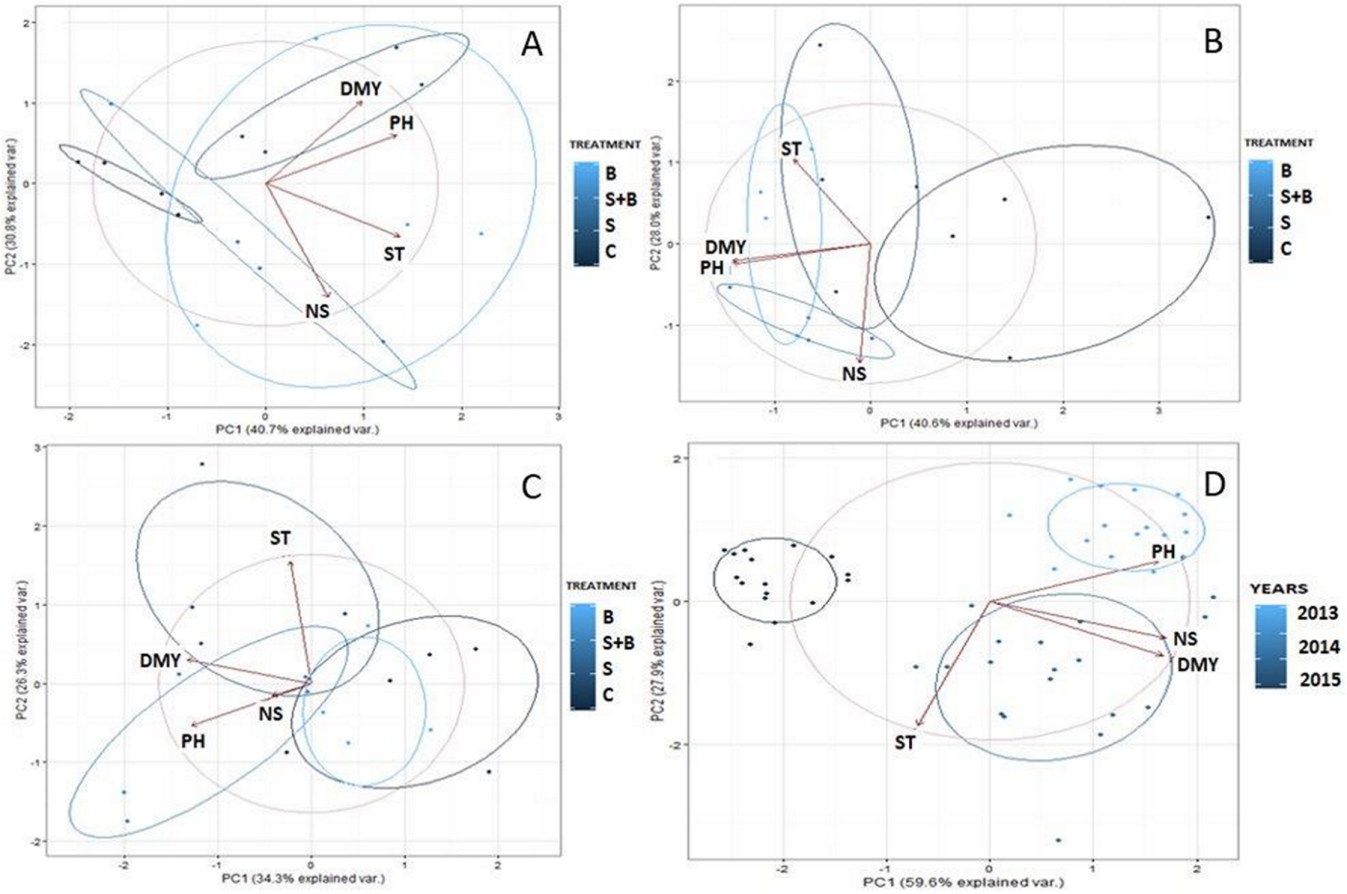
Principal component analysis (PCA) for willow according to treatments: C, control; S, sewage sludge; S+B, sewage sludge + BAF; B—BAF. (A) 3 months after application. (B) 15 months after application. (C) 27 month after application. (D) Analyses of the biometric parameters in the years of the study. Abbreviation: DMY, dry matter yield; ST, shoot thickness; PH, plant height; NS, number of shoot.

Biometric characteristics and dry mass of willow plants were subjected to PCA to underline differences between the tested treatments. The analysis of our results shows that the components PC 1 and PC 2 explained 71% (A), 69% (B), 60% (C) and 88% (D) of the data set variation. In the first year, positive relations were noted in the pairs NS and ST, and DMY and PH. The control plants were negatively related to examined biometrical parameters (Fig. 6A). In the second year a high positive association between DMY and PH was recorded, furthermore variants sludge sewage and BAF and alone BAF were positively related to all biometrical parameters (Fig. 6B). In the third year a positive relation between almost all analysed biometrical parameters and variant with sludge sewage and BAF was found (Fig. 6C). The PCA throughout all years revealed a positive relation between NS and DMY, as well as between all analysed treatments and biometrical parameters. This was also valid for the third year (excluding ST) (Fig. 6D).

## Discussion

Microbiological indexes included in the analyses of the soil environment following application of various fertilisers, for example, chemically stabilized sewage sludge, reflect the ecological status of soil (Lloret et al., 2016; Napora & Grobelak, 2014; Thangarajan et al., 2013). An increase in the number of certain microbial groups, for example, bacteria, fungi and Actinobacteria, is significantly related to the level of organic carbon and the C:N ratio observed following sewage sludge application (Jezierska-Tyś & Frąc, 2005; Martí et al., 2015). A significant problem is also connected with the assessment of a long-term effect of municipal sewage sludge application on the functional diversity of microbial communities in the soil environment (Chakraborty et al., 2011; Smalla & Van Elsas, 2010).

The highest total number of bacteria and Actinobacteria were recorded in the second year of sludge influence. Also results reported by Furczak & Joniec (2007a) showed that the positive effect of sludge is not short-term and the increase in counts of bacteria and filamentous fungi is maintained up to 2 years after sludge application. Those authors stressed that microorganisms found in sludge do not colonise the soil environment and within a short period after sludge introduction to soil most of them die, thus constituting an additional source of organic matter. Literature data indicate that an increase in bacterial number may be promoted by other advantageous changes in the soil environment, that is, elevated pH and improved air and water relations following sewage sludge application (Furczak & Joniec, 2007b). Next to Eubacteria and Actinobacteria, the primary role in mineralisation of organic matter in soil is attributed to filamentous fungi (Wolna-Maruwka et al., 2014a).

Literature sources on the subject include reports that sludge application to soil causes an increase in counts not only of bacteria and Actinobacteria but also of fungi (Marschner, Kandeler & Marschner, 2003). In their study Nowak, Kacprzak & Grobelak (2010) showed that sewage sludge application increased counts of fungal populations, that is, *Penicillium, Verticillium, Mucor, Mortierella, Fusarium, Geotrichum* and *Trichoderma*, among which the latter is an antagonist of many plant pathogens. In this experiment an increase was also recorded in the population of the discussed microbial group in relation to the control following application of the BAF alone. In a study by Wolna-Maruwka et al. (2014a, 2014b) it was also found that the BAF, irrespective of the inoculation method or application rate, stimulated growth of mould fungi in the peat substrate. A reduction of fungal counts in the year after sludge application could have been connected in this study with the restoration of equilibrium in the soil environment and depletion of scarcely degradable compounds, in decomposition of which these fungi are pioneering organisms.

Stimulation of microbial growth is also accompanied by the stimulation of soil biochemical activity. Introduction of sludge to soil causes marked changes in enzymatic activity, while the direction and intensity depend on the type of sludge, its application rates and the enzyme (Napora & Grobelak, 2014; Selivanovskaya & Latypova, 2006; Yazdanpanah, Mahmoodabadi & Cerdà, 2016). Changes in the activity of soil enzymes reflect disturbances in the environment affecting both the soil and plants (Bielińska & Mocek-Płóciniak, 2012). The most frequently investigated soil enzymes include dehydrogenases as well as acid and alkaline phosphatase (Bielińska & Mocek-Płóciniak, 2009).

In our experiments the applied factors had a significant effect modifying levels of the the DHA in lessive soil. Literature data indicate that the activity of these enzymes increases with an increase in the amount of organic matter introduced to soil (Jezierska-Tyś & Frąc, 2008). In a study conducted for three years, in intensive farm under greenhouse conditions, Morra et al. (2010) used different doses of compost (15, 30, 45 t ha^−1^) and compost (at a dose of 15 t ha^−1^) combined with mineral N fertiliser to investigate the effects of exogenous organic matter on soil enzymatic activities. They found that soil respiration, fluorescein diacetate hydrolase and phosphomonoesterase activities increased after compost application. It is assumed that among the analysed parameters of soil enzymatic activity PAL is a good indicator of soil microorganisms modifying soil productivity, that is, through mineralisation of organic phosphorus compounds (Bielińska, Futa & Mocek-Płóciniak, 2014; Rahmansyah, Antonius & Sulistinah, 2009).

When determining acid phosphatase activity in the soil we need to remember that plants have a considerable effect on the level of this enzyme in the soil environment, as their roots secrete large amounts of acid phosphatase, particularly in the case of phosphorus deficit (Scotti et al., 2015; Żebrowska & Ciereszko, 2009). Higher concentrations of acid phosphatase in the control variant—in relation to the treatments with the application of sludge and the inoculant—may have been the response of plants to phosphorus levels in the soil environment.

The results of our study indicate that the limitation or stimulation of microbial activity was closely related to the availability of organic matter. Sewage sludge following its application to soil becomes a source of available organic carbon and macronutrients, thanks to which they stimulate microbial growth and enzymatic activity. Similar relationships of microbiological and biochemical activity were noted for nitrogen transformation analyses (Wolna-Maruwka et al., 2018). The authors noted that in relation to the control object all combinations used with the addition of sewage sludge contributed to a significant increase in the assimilation area of leaves and the nutritional status of plants with nitrogen (SPAD). Research indicates the stimulating effect of sewage sludge on the multiplication and activity of proteolytic bacteria and urease activity. Moreover, a positive relationship between the nitrogen nutrient status of plants—SPAD and the plant leaf assimilation plant ratio and the urease activity level was demonstrated.

Sewage sludge revealed a positive effect on plant growth and has been widely used in agriculture and horticulture for cultivation of several plant species (Antolín et al., 2005; Pascual et al., 2004). This is a donor of additional *P* and *N* source for plants. Moreover, some sewage sludge components are necessary for proper growth of soil microorganisms, and can improve the microbiological status of the soil (Pascual et al., 2007; Singh & Agrawal, 2008) and in turn exert a positive effect on plants and their photosynthetic intensity. Some authors have found that leaf protein content increased (as indicated by *N* concentration) in sludge-treated plants (Mata-González, Sosebee & Wan, 2004). There is low number of investigations on synergistic effect of sewage sludge and effective microorganisms on plant photosynthesis. The most of investigations carried out on positive effect of sewage sludge application on photosynthesis activity of selected plant species, such as *Medicago sativa* (Baioui et al., 2017), maize, sunflower, barely (Singh & Agrawal, 2008), The positive effect on photosynthesis activity is mostly relate to increased radiation use efficiency involved in CO_2_ assimilation, together with stimulation of coefficient of photochemical quenching and transpiration (Lakhdar et al., 2011). Our investigations are in agreement with these findings, as increase of *P*_N_ and *E* were found, for both BAF and sewage sludge treatment. It was also previously found that sewage sludge positively influenced plants treated with water deficit, through higher rates of photosynthesis and lower intercellular CO_2_ (Antolín, Muro & Sánchez-Díaz, 2010).

Willow is a species responding strongly to environmental conditions, particularly organic and mineral fertilisation (Szczukowski et al., 2004). The application of sewage sludge in this study significantly modified the yield of willow dry mass. An advantageous effect on yield volume was found after the application of both sludge and the BAF. Results reported by many authors indicate an increase in yields with the passing years of the experiments (Jurczuk, Chrzanowski & Jaszczyński, 2010); thus frequently biomass is harvested in a 1-, 2- or 3-year rotation cycle. The low yield obtained in the third year of culture resulted from worse moisture conditions in 2015. An advantageous effect of sewage sludge application was also reported by Czyż & Kitczak (2014), Holm & Heinsoo (2013) and Sobczyk et al. (2015). Stańczyk-Mazanek, Bień & Kępa (2014) harvested the greatest volume of willow after fertilisation with sewage sludge at 10 and 50 Mg·ha^−1^. In turn, Heinsoo & Dimitriou (2014) when evaluating the effects of sewage sludge application in three locations, that is, Estonia, Germany and Poland, found no significant effect of that factor on an increase in willow biomass, while they only indicated habitat diversification.

In our experiments the thickest rods were observed not in the first year but in the following 2 years in treatments with sewage sludge application. Similarly, Sobczyk et al. (2015) observed a considerable increase in rod thickness in two willow clones, 1,056 and 1,059, as a result of sewage sludge fertilisation. Czyż & Kitczak (2014) in their study on the response of willow to compost fertilisation also observed a positive effect of that factor on ST and length as well as DMY.

The application of sewage sludge had no significant effect on variation in NSs from a rootstock, however, an upwards trend may be observed in relation to the control for each of the tested combinations. Investigations conducted by Adams, De-Leij & Lynch (2007) on the effects of *Trichoderma* spp. application in willow culture indicate effective stimulation of growth and development of this species due to changes in soil activity.

## Conclusions

Application of sewage sludge increased yield and selected biometric traits of willow plants. Among the tested combinations the best results were obtained following the introduction of sewage sludge together with the BAF. The most advantageous effects of sewage sludge as well as sludge applied together with the BAF prior to the establishment of the willow plantation was observed in the second year of the study, which was manifested in the case of dry mass yield, ST, the NSs from a rootstock as well as PH. Similar dependencies were observed for the efficiency of photosynthesis and soil microbial activity.

## Supplemental Information

10.7717/peerj.6434/supp-1 Supplemental Information 1 Raw data.

## Additional Information and Declarations

### Data Availability

The following information was supplied regarding data availability: The raw data are available in a Supplemental File.
